# The Effect of Long-Term Betacoronavirus Infection on the Permeability of the Blood–Brain Barrier—In Vitro Model Studies

**DOI:** 10.3390/cells14191493

**Published:** 2025-09-24

**Authors:** Weronika Daria Krahel, Marcin Chodkowski, Michalina Bartak, Agnieszka Ostrowska, Michał M. Godlewski, Maksymilian Adamczyk, Małgorzata Krzyżowska, Joanna Cymerys

**Affiliations:** 1 Division of Microbiology, Department of Preclinical Sciences, Institute of Veterinary Medicine, Warsaw University of Life Sciences, 02-786 Warsaw, Poland; michalina_bartak1@sggw.edu.pl (M.B.); maksymilian_adamczyk@sggw.edu.pl (M.A.); 2 Division of Medical and Environmental Microbiology, Military Institute of Hygiene and Epidemiology, 01-163 Warsaw, Poland; marcin.chodkowski@wihe.pl (M.C.); malgorzata.krzyzowska@wihe.pl (M.K.); 3 Department of Nanobiotechnology, Institute of Biology, Warsaw University of Life Sciences, 02-786 Warsaw, Poland; agnieszka_ostrowska@sggw.edu.pl; 4 Department of Physiological Sciences, Institute of Veterinary Medicine, Warsaw University of Life Sciences, 02-776 Warsaw, Poland; michal_godlewski@sggw.edu.pl

**Keywords:** blood–brain barrier, MHV-JHM, coronavirus, mitochondria, reactive oxygen species, F-actin

## Abstract

The blood–brain barrier (BBB) is critical for central nervous system homeostasis, yet it is highly vulnerable to viral insults. While acute coronavirus infections are known to impair BBB integrity, the long-term impact of persistent infection on brain endothelial cells remains poorly understood. Using an in vitro BBB model, we examined the effects of a 12-week infection with the neurotropic murine coronavirus MHV-JHM. Structural and functional changes were assessed via fluorescein isothiocyanate (FITC)-dextran permeability assay, confocal imaging of mitochondria, actin cytoskeleton, reactive oxygen species (ROS), scanning electron microscopy (SEM) and RT-qPCR for viral RNA level. Long-term infection induced progressive mitochondrial fragmentation and sustained ROS overproduction. Permeability to 70 kDa dextran increased significantly at 48 h post-infection and exceeded control levels threefold by 168 h. SEM revealed gradual endothelial surface roughening, blebbing, and eventual monolayer collapse with extensive intercellular gaps by week 12. Our study demonstrates that long-term MHV-JHM infection profoundly alters brain endothelial cell structure and function, triggering a cascade of changes that culminate in the disintegration of the BBB model.

## 1. Introduction

The blood–brain barrier (BBB) is a highly specialised and dynamic interface that regulates the exchange of molecules, ions, and immune cells between the bloodstream and the central nervous system (CNS), playing a pivotal role in maintaining neural homeostasis. It is composed primarily of brain microvascular endothelial cells (BMECs), which are joined by continuous tight junctions (TJs) and adherens junctions, possess minimal transcytosis capacity, and express a range of selective transporters and efflux proteins [1]. These endothelial cells are supported by pericytes embedded in the basement membrane and astrocyte end-feet that ensheath the capillaries, forming the neurovascular unit (NVU), a functional complex essential for BBB maintenance and signalling [2]. Functionally, the BBB serves as both a physical and metabolic barrier. It limits the entry of potentially neurotoxic compounds and pathogens while enabling the controlled transport of nutrients, hormones, and metabolic waste. This barrier also plays a central role in neurovascular coupling, aligning blood flow with neuronal metabolic demand [3]. The selective permeability of the BBB is achieved through the expression of tight junction proteins (e.g., claudins, occludin, and ZO-1) and a variety of transporters, including GLUT1, LAT1, and P-glycoprotein, which regulate the bidirectional movement of solutes. Importantly, the BBB is not a static structure. Its integrity and function are dynamically regulated during development and are responsive to environmental cues, inflammatory stimuli, and disease states [4]. Disruption of the BBB has been implicated in a range of neuropathologies, including multiple sclerosis, stroke, epilepsy, and neurodegenerative diseases such as Alzheimer’s disease. Such disruption often involves oxidative stress, inflammatory cytokine release, cytoskeletal remodelling, and degradation of tight junction proteins—processes that collectively increase permeability and lead to leukocyte infiltration and oedema formation [1].

One of the central mechanisms contributing to BBB dysfunction in disease is oxidative stress, particularly that mediated by NADPH oxidase (Nox) enzymes. Nox-derived reactive oxygen species (ROS), especially superoxide and hydrogen peroxide, can initiate endothelial damage by degrading TJ proteins, activating matrix metalloproteinases (MMPs), and promoting cytoskeletal contraction. For example, in experimental stroke models, Nox2 and Nox4 have been shown to induce ROS production that correlates with BBB permeability and infarct size. Pharmacological inhibition or genetic deletion of these enzymes results in better barrier preservation and improved outcomes [5,6,7]. Furthermore, proinflammatory cytokines such as TNF-α and IL-17 act through Nox to impair tight junctions and downregulate the endothelial phenotype [8,9]. These effects are compounded by age-related reductions in antioxidant defences and regulatory proteins, including Sirt1 and Sirt3, which are known to modulate mitochondrial redox homeostasis and endothelial survival [10,11].

Mitochondria represent an additional, potent source of ROS, especially under stress conditions such as hypoxia, ischemia, or exposure to neurotoxic agents. Mitochondrial fragmentation, driven by overactivation of dynamin-related protein 1 (Drp1), has been implicated in endothelial barrier loss [12]. Concurrently, impaired mitophagy, through dysregulation of PINK/Parkin signalling, leads to the accumulation of dysfunctional mitochondria and exacerbates ROS burden [13]. Such mitochondrial disturbances can initiate reorganisation of the actin cytoskeleton, a key component of BBB integrity. Activation of RhoA and its downstream effector ROCK, often in response to intracellular calcium or oxidative stress, triggers phosphorylation of myosin light chain kinase (MLCK), resulting in actomyosin contraction, stress fiber formation, and endothelial tightening. This mechanical strain on the cytoskeleton disrupts intercellular junctions and promotes paracellular leakage [14,15]. Numerous studies have shown that various stimuli, including methamphetamine, methylglyoxal, β-amyloid peptides, and advanced glycation end-products, alter actin architecture and increase BBB permeability via this pathway [13,16,17].

Hypoxia/reoxygenation injury further exemplifies the convergence of oxidative stress and actin dynamics in BBB failure. During reoxygenation, surges in ROS levels disrupt TJ protein expression, degrade occludin and ZO-1, and induce pronounced F-actin rearrangement. These changes occur in tandem with increased permeability to solutes and immune cells [18,19]. Moreover, the use of Rho kinase inhibitors or MLCK blockers in such models has been shown to restore barrier function partially, highlighting the importance of cytoskeletal regulation [20,21].

Viral infections also pose a substantial threat to BBB integrity. Recent clinical evidence indicates that human coronaviruses, including SARS-CoV-2, affect BBB integrity and CNS function. COVID-19 patients frequently presented neurological symptoms such as anosmia, encephalopathy, stroke, and neurocognitive deficits [22,23,24]. Biomarkers of BBB disruption, such as elevated S100B and GFAP levels, have been observed early and persistently in infected patients with neurological manifestations [25,26]. Mechanistic studies suggest that neuroinflammation, microvascular injury, and matrix metalloproteinase activation are major contributors to coronavirus-induced BBB damage [27]. While some findings argue against direct viral invasion of the brain parenchyma [28], others support neurotropism and BBB involvement via endothelial infection, cytokine storm, or immune cell trafficking [29,30,31]. In support of a possible direct effect of the virus on the brain, some studies have shown: (i) the presence of SARS-CoV-2 mRNA in the cerebrospinal fluid of patients with acute COVID-19 [32,33], (ii) the presence of angiotensin converting enzyme-2 (ACE2) receptors on glial cells, which could be an entry pathway for SARS-CoV-2, (iii) the presence of ACE2 receptors in the blood–brain barrier (BBB) [34,35], and (iv) altered BBB integrity and reduced blood flow, immune cell infiltrations, as well as the presence of SARS-CoV-2 in cortical neurons of patients who died from COVID-19 [36,37,38,39,40]. 

Most of these findings have only been reported in the acute stages of SARS-CoV-2 infection, and as these changes may be either direct or indirect, it is still unclear how viral infection affects the brain over the longer term. The increasing prevalence of long-term neurological complications resulting from SARS-CoV-2 infection calls for intensified research into the neurotropism and neuropathogenicity of coronaviruses. To achieve this, it is crucial to establish in vitro models that are appropriate for studying the molecular mechanisms of coronaviral neuropathogenicity. Murine hepatitis virus (MHV), a member of the betacoronavirus genus, has long been used as a model of CNS infection [41,42,43,44,45]. Studies with MHV-3 have demonstrated loss of ZO-1, occludin, and VE-cadherin in infected endothelial monolayers, an effect not reversed by neutralising cytokines, suggesting direct viral cytopathic action [46,47]. Analyses have confirmed that MHV-JHM can induce loss of BBB function, in part via MMP activation and cytokine cascades [48].

We raise questions on the potential long-term effects of coronaviral infection on the blood–brain barrier integrity. In the current study, we investigated the effects of long-term (12-week) infection with MHV-JHM in the murine brain endothelial cell line bEnd.3. We focused on the interplay between prolonged viral stress, mitochondrial dynamics, ROS generation, and cell membrane remodelling, which could be a potential mechanism of BBB disruption, with long-term implications for CNS homeostasis.

## 2. Materials and Methods

### 2.1. Cell Cultures

The bEnd.3 cell line (mouse brain endothelial cells), Neuro-2a cell line (mouse neuroblasts) and C8-D1A cell line (mouse astrocytes) were used in the study (CRL-2299™, CCL-131™, CRL-2541™, respectively, ATCC^®^, Manassas, CA, USA). Cell cultures were grown in DMEM (Dulbecco’s Modified Eagle Medium with GlutaMAX™ Supplement, sodium pyruvate and 4.5 g/L D-Glucose) supplemented with 10% fetal bovine serum (FBS) and 1% Penicillin-Streptomycin (10,000 U/mL) (Gibco™, Waltham, MA, USA). Cell cultures were incubated at 37 °C with 5% CO_2_. All experiments were conducted when the cell confluence reached 90–100%.

### 2.2. Transwell System

**Figure 1:**
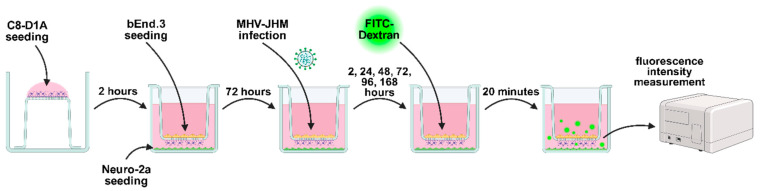
A triple cell co-culture model of bEnd.3, Neuro-2a and C8-D1A cells used in the FITC-Dextran permeability assay. Created in BioRender. Krahel, W. (2025) https://BioRender.com/3zbgf3r.

To assess the BBB permeability, 24-well tissue culture inserts with 1 µm pores (Sarstedt, Nümbrecht, Germany) were used in a co-culture system (bEnd.3, Neuro-2a and C8-D1A) (Figure 1). C8-D1A cell line was seeded on the basolateral side of the filter membrane. After incubation (2 h, 37 °C, 5% CO_2_), bEnd.3 cell line was seeded on the apical side of the filter membrane, and the Neuro-2a cell line was seeded on the receiver 24-well plate [49]. Each cell line in the co-culture system was seeded at 103 cells/cm2 density. Cells were grown in a culture medium described above, with 800 µL medium in the receiver plate well and 300 µL of medium in the insert. Cell cultures were incubated at 37 °C with 5% CO_2_. All experiments were conducted when the cell confluence reached 100%.

### 2.3. Virus

**Figure 2:**
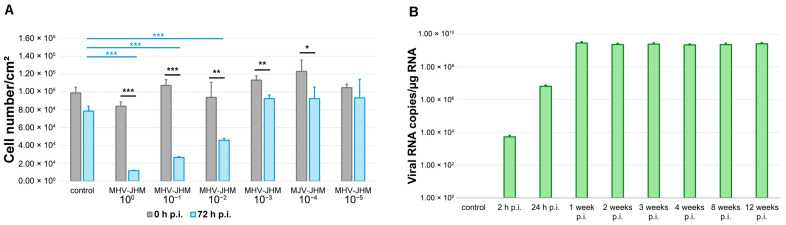
MHV-JHM replication in bEnd.3 cell line. Number of bEnd.3 cells per cm^2^ at 0 and 72 h after infection with 10-fold dilutions of MHV-JHM stock (**A**). Control—uninfected cells; h p.i.—hour post-infection. Data is shown as the mean number of cells/cm^2^ with SD. Kruskal–Wallis test with Dunn’s post hoc; * *p* < 0.05, ** *p* < 0.01, *** *p* < 0.001. Black asterisks—statistically significant changes between the start and end of observation at given virus dilution. Blue asterisks—statistically significant changes in cell number/cm^2^ between control and 10-fold virus dilution at 72 h p.i. RT-qPCR analysis of MHV-JHM RNA copies per µg RNA during 12 weeks of infection in bEnd.3 cells (**B**). Data shown as a mean number of copies ± SD. Week p.i.—week post-infection.

Murine hepatitis virus (MHV), neuropathological strain MHV-JHM (VR-765™, ATCC^®^, Manassas, CA, USA) was propagated in an NCTC clone 1469 (CCL-9.1, ATCC^®^, Manassas, CA, USA) cell line. The median tissue culture infectious dose (TCID_50_) was calculated using the Spearman-Kärber method (TCID_50_/mL = 10^7.8^) [50]. Aliquots were stored at −70 °C. HoloMonitor^®^ PHI M4 (Phase Holographic Imaging, Lund, Sweden) was used to perform label-free, real-time monitoring of bEnd.3 cells over 72 h following infection with 10-fold dilutions of MHV-JHM stock (10^0^–10^−5^). Uninfected bEnd.3 cells were used as a control. Quantification of changes in the cell number was conducted. Results were used to select the optimal virus dilution (10^−3^) for subsequent experiments (Figure 2).

### 2.4. Infection of Cell Cultures with MHV-JHM

bEnd.3 cell line in multiple well plates and in co-culture Transwell systems were infected MHV-JHM at MOI = 0.005. After adsorption (1 h, 37 °C, 5% CO_2_), the virus suspension was discarded, and fresh growth medium was added. Cell cultures in multiple well plates were incubated for 2, 24 h, and 1, 2, 3, 4, 8, 12 weeks (37 °C, 5% CO_2_). Cell cultures in Transwell systems were incubated for 2, 24, 48, 72, 96, and 168 h (37 °C, 5% CO_2_). Half of the culture medium was replaced with a fresh medium every 2–3 days starting from the first week of infection to maintain cells for extended time points post-infection.

### 2.5. FITC-Dextran Permeability Assay

To assess the permeability of bEnd.3 monolayer after MHV-JHM infection (2, 24, 48, 72, 96, 168 h), a Transwell system with bEnd.3, C8-D1A and Neuro-2a co-culture was used (Figure 1). Uninfected cultures were used as negative controls. Each control was incubated for the same time as the respective infected culture. Culture medium was carefully removed from each insert and receiver plate well. Fresh culture medium was added to the receiver plate wells (800 µL). Fluorescein isothiocyanate (FITC)-dextran (70 kDa; ThermoFisher™, Waltham, MA, USA) was diluted in fresh culture medium and added to inserts, allowing the permeation of the monolayer (10 µg/mL, 300 µL, 20 min, 37 °C, in the dark). Inserts were transferred from the receiver wells into fresh plate for fixation with PFA/PBS (3.7%, 15 min, RT) prior to further fluorescence staining. Medium in receiver wells was thoroughly mixed and removed from each well to a 96-well plate (100 µL in triplicate) for fluorescence measurement. The fluorescence was measured with a Synergy H1 plate reader (BioTek™, Winooski, VT, USA) using 494 and 521 nm as the excitation and emission wavelengths, respectively. The quantity of FITC-dextran permeated through a monolayer was calculated using a standard curve (0.5, 0.25, 0.1, 0.01, 0.001 µg/mL FITC-dextran, blank—culture medium) [51].

### 2.6. Immunofluorescence Staining

#### 2.6.1. F-Actin and Viral Antigen

F-actin filaments in bEnd.3 cell line in the Transwell system were stained at 2, 24, 48, 72, 96 and 168 h post-infection (h p.i.). Uninfected bEnd.3 cells in the Transwell system were used as a negative control. Cell cultures were fixed with PFA/PBS (3.7%, 15 min, RT) and washed twice with PBS. The insert membranes were removed after fixation and placed in a 24-well plate for staining. Next, cells on membranes were incubated with Tween/PBS (1%, 5 min, RT), washed twice with PBS and incubated with BSA/PBS (1%, 20 min, RT). To visualize MHV-JHM antigen, cells were incubated with SARS/SARS-CoV-2 Spike Protein S2 Monoclonal Antibody (1:250, overnight, 4 °C; ThermoFisher™, Waltham, MA, USA), washed thrice with PBS and incubated with Goat anti-Mouse IgG (H + L) Superclonal™ Secondary Antibody, Alexa Fluor™ 488 (1:500, 1 h, RT, in the dark; ThermoFisher™, Waltham, MA, USA). Cells were washed thrice with PBS and incubated with Phalloidin-TRITC (500 µg/mL, 1 h, RT, in the dark; Sigma-Aldrich^®^, Burlington, MA, USA) to visualise F-actin filaments. Cells were washed thrice with PBS, and cell nuclei were counterstained with Hoechst 33,258 (1 µg/mL, 2 min, RT; ThermoFisher™, Waltham, MA, USA). Cells were then washed twice with PBS and once with dH_2_O, then mounted on glass slides with Prolong Gold Antifade Reagent (ThermoFisher™, Waltham, MA, USA).

#### 2.6.2. Mitochondria and Viral Antigen

Changes in the mitochondrial network in bEnd.3 cell line (3 × 10^6^ cells per well on 6-well plates) were assessed 2, 24 h p.i. and 1, 2, 3, 4, 8, 12 weeks p.i. Uninfected cells were used as a negative control. Culture medium was discarded, and cells were stained with Mito Red (Sigma-Aldrich^®^, Burlington, MA, USA) diluted in fresh culture medium (100 nM, 30 min, 37 °C). Cells were washed twice with PBS and fixed with PFA/PBS (3.7%, 15 min, RT). After fixation, cells were washed twice with PBS and incubated with Tween/PBS (1%, 5 min, RT). Next, they were washed twice with PBS and incubated with BSA/PBS (1%, 20 min, RT). To visualize MHV-JHM antigen, cells were incubated with SARS/SARS-CoV-2 Spike Protein S2 Monoclonal Antibody (1:250, overnight, 4 °C; ThermoFisher™, Waltham, MA, USA), washed thrice with PBS and incubated with Goat anti-Mouse IgG (H + L) Superclonal™ Secondary Antibody, Alexa Fluor™ 488 (1:500, 1 h, RT, in the dark; ThermoFisher™, Waltham, MA, USA). Cells were then washed thrice with PBS, and cell nuclei were counterstained with Hoechst 33,258 (1 µg/mL, 2 min, RT; ThermoFisher™, Waltham, MA, USA). Afterwards, cells were washed twice with PBS and once with dH_2_O, then mounted on glass slides with Prolong Gold Antifade Reagent (ThermoFisher™, Waltham, MA, USA).

#### 2.6.3. Reactive Oxygen Species (ROS)

ROS levels in bEnd.3 cell line (3 × 10^6^ cells per well on 6-well plates) were assessed 2, 24 h p.i. and 1, 2, 3, 4, 8, 12 weeks p.i. Uninfected cells were used as a negative control. Uninfected cells treated with 1% H_2_O_2_ (1 min, RT) were used as a positive control. Cell culture medium was discarded, and fluorogenic probe CellROX^®^ Green Reagent (ThermoFisher™, Waltham, MA, USA) diluted in fresh culture medium was added to cells (5 µM/mL, 30 min, 37 °C). Cells were washed twice with PBS and fixed with PFA/PBS (3.7%, 15 min, RT). After fixation, cells were washed twice with PBS and cell nuclei were stained with Hoechst 33,258 (1 µg/mL, 2 min, RT; ThermoFisher™, Waltham, MA, USA). Cells were washed twice with PBS and once with dH_2_O, then mounted on glass slides with Prolong Gold Antifade Reagent (ThermoFisher™, Waltham, MA, USA). 

### 2.7. Confocal Imaging and Analysis

Images of stained cell cultures were acquired using a confocal microscope Fluoview FV10i (Olympus, Tokyo, Japan), and saved in 24-bit.tiff format, and analysed using FV10i software version 4.1 (Olympus, Tokyo, Japan). ROS levels were analysed using ImageJ (NIH Image, version 1.53a, USA) [52]. Mitochondrial network was analysed using ImageJ (NIH Image, version 1.53a, USA) and MiNa Single Image macro tools, allowing determination of point mitochondria (individuals), cross-linked mitochondria (network), mean branch length [µm], mean network size [µm], and mitochondrial area in the cell (mitochondrial footprint [µm^2^]) [53]. Confocal microscopy images of 300 cells at each time point were used for ROS level analysis, and 100 cells at each time point for the mitochondrial network analysis.

### 2.8. Scanning Electron Microscopy (SEM)

bEnd.3 cells on coverslips (3 × 10^5^ cells per well in 24-well plate), after 1, 2, 4, 8 and 12 weeks of infection, were fixed for 2 h with 2.5% glutaraldehyde in phosphate buffer. Uninfected cells were used as a control. Next, the coverslips were incubated with phosphate buffer for 2 h and post-fixed for 1 h in 1% OsO_4_. After washing with dH_2_O, samples were dehydrated through a series of increasing concentrations (30–100%) of ethanol and acetone (2 × 30 min) and dried in a CPD 7501 critical point drier (Polaron; Hatfield, PA, USA). Cells were then coated with a gold layer in a JFC-1300 sputter-coater (JEOL, Tokyo, Japan). The SEM imaging was conducted under a FEI Quanta 200 environmental scanning electron microscope (ESEM) with an EDAX EDS system (FEI, Tokyo, Japan).

### 2.9. RNA Extraction and Reverse Transcription Quantitative Real-Time PCR (RT-qPCR)

RNA was extracted from bEnd.3 cells (3 × 10^6^ cells per well in 6-well plate) using GeneMATRIX Universal DNA/RNA/Protein Purification Kit (EURx^®^, Gdansk, Poland) according to the protocol provided by the manufacturer and eluted in 50 µL of RNase-free water. Real-Time RT-qPCR was performed using the QunatiNova RT-qPCR Kit (Qiagen Inc., Santa Clarita, CA, USA) on a thermocycler QuantStudio 5 (Applied Biosystems, Foster City, CA, USA). Standard RT-qPCR was performed according to the manufacturer’s recommendations. 500 ng of extracted RNA was mixed with 2 µL Probe RT-PCR MasterMix, 6 μL of RNase-free water, 1.5 µL of each primer: MHV-F (5′–GGA ACT TCT CGT TGG GGC ATT ATA CT–3′), MHV-R (5′–ACC ACA AGA TTA TCA TTT TCA CAA CAT A–3′), 1 µL of Probe 5′ 6-FAM 3′ TAMRA (5′-6-FAM-ACA TGC TAC GGC TCG TGT AAC CGA ACT GT–TAMRA-3′) and 0.2 µL QN Probe RT-Mix, and filled with water to a total volume of 20 µL. The following cycle conditions were used: RT-step at 45 °C, 10 min, PCR initial heat activation at 95 °C, 5 min, and 40 cycles of 2-step cycling (denaturation at 95 °C, 5 s and combined annealing at 60 °C, 30 s). Primers were designed based on the region encoding membrane protein (nucleotides 29196–29303; product length 108 nt) in the MHV-JHM genome sequence (GenBank: FJ647226.1). Primers were purchased from IBB PAN (Warsaw, Poland). A standard curve was prepared using serial 10-fold dilutions of pGEM-T/MHV-JHM standard (10^6–10^ copies per reaction). Each reaction was performed in a triplicate.

### 2.10. Statistical Analysis

Normality of data was evaluated using the Shapiro–Wilk test. Homogeneity of variances was assessed using the Levene test and the Brown–Forsythe test. The results were statistically evaluated using the Kruskal–Wallis test and post hoc Dunn’s test. All experiments were performed in a triplicate. Analyses were performed using Statistica version 13.3 (TIBCO Software Inc., Palo Alto, CA, USA). Statistical differences were interpreted as significant (*) at *p* ≤ 0.05, highly significant (**) at *p* ≤ 0.01, extremely significant (***) at *p* ≤ 0.001, and insignificant at *p* > 0.05.

## 3. Results

### 3.1. MHV-JHM Replication in bEnd.3 Cell Line

To identify a viral dose suitable for establishing long-term, non-cytolytic MHV-JHM infection in bEnd.3 cells, we monitored the proliferation of cultures infected with serial 10-fold dilutions of virus stock (10^0^ to 10^−5^) over 72 h using HoloMonitor^®^ PHI M4 (Phase Holographic Imaging, Lund, Sweden) live-cell imaging. Uninfected controls were included for comparison. Cell counts were recorded at 0 h and 72 h p.i., and statistical analyses were performed to assess both within-group changes and differences between conditions (Figure 2A).

In the control group, cell number per cm^2^ decreased slightly, but not significantly, over 72 h (from 98,860 ± 6443 (mean ± SD) to 88,418 ± 5396; *p* = 0.1), likely reflecting contact inhibition in confluent cultures. By contrast, cultures infected with undiluted virus (10^0^) showed a significant decrease in cell number per cm^2^ (from 84,051 ± 4699 to 11,681 ± 584; *p* < 0.001), indicating severe cytopathic effect. A similar effect was observed at the 10^−1^ dilution (from 107,231 ± 6387 to 26,477 ± 1006; *p* < 0.001). The 10^−2^ dilution also led to a significant reduction in cell number per cm^2^ (from 93,837 ± 16,850 to 45,789 ± 2152; *p* = 0.009), while the 10^−3^ dilution resulted in sustained growth with moderate reduction (from 113,189 ± 4791 to 92,552 ± 3953; *p* = 0.017). Higher dilutions (10^−4^ and 10^−5^) did not significantly alter cell numbers (from 122,988 ± 12,628 to 92,552 ± 12,830, *p* = 0.066 and from 104,739 ± 3855 to 93,331 ± 20,759, *p* = 0.2445, respectively).

A comparison of final cell number counts per cm^2^ between control and infected cells at 72 h resulted in significant differences between control and undiluted virus (*p* < 0.001), 10^−1^ dilution (*p* < 0.001), and 10^−2^ dilution (*p* < 0.001) (Figure 2A).

MHV-JHM 10^−3^ dilution produced a significantly lower final cell count compared to the starting point, but no significant changes were detected compared to the final cell count in the control (*p* = 1). These findings indicated using the 10^−3^ dilution of MHV-JHM for further experiments, as it induced a measurable biological effect without causing widespread cell death (Figure 2A).

The number of viral RNA copies in bEnd.3 cells during long-term infection was determined (Figure 2B). At 2 h p.i., the number of copies was 5.3 × 10^3^ ± 1.3 × 10^3^ (mean ± SD) per µg RNA. Then, at 24 h p.i. values increased to 6.5 × 10^6^ ± 1.1 × 10^6^ copies/µg RNA when the complete replication cycle occurred. At 1 week p.i., values rose to 2.9 × 10^9^ ± 0.5 × 10^9^, and slightly decreased in the following times post-infection (2.3 × 10^9^ ± 0.4 × 10^9^ at 2 weeks p.i.; 2.5 × 10^9^ ± 0.5 × 10^9^ at 3 weeks p.i.; 2.2 × 10^9^ ± 0.3 × 10^9^ at 4 weeks p.i.; 2.3 × 10^9^ ± 0.5 × 10^9^ at 8 weeks p.i.; 2.6 × 10^9^ ± 0.4 × 10^9^ at 12 weeks p.i.).

### 3.2. Endothelial Barrier Permeability in MHV-JHM-Infected Triple Co-Culture BBB Model

To assess the impact of MHV-JHM infection on endothelial barrier integrity, we performed a FITC-dextran permeability assay on bEnd.3 monolayers in a triple co-culture Transwell system at multiple time points post-infection. Uninfected control cultures processed in parallel served as a time-matched references for the baseline permeability.

**Figure 3:**
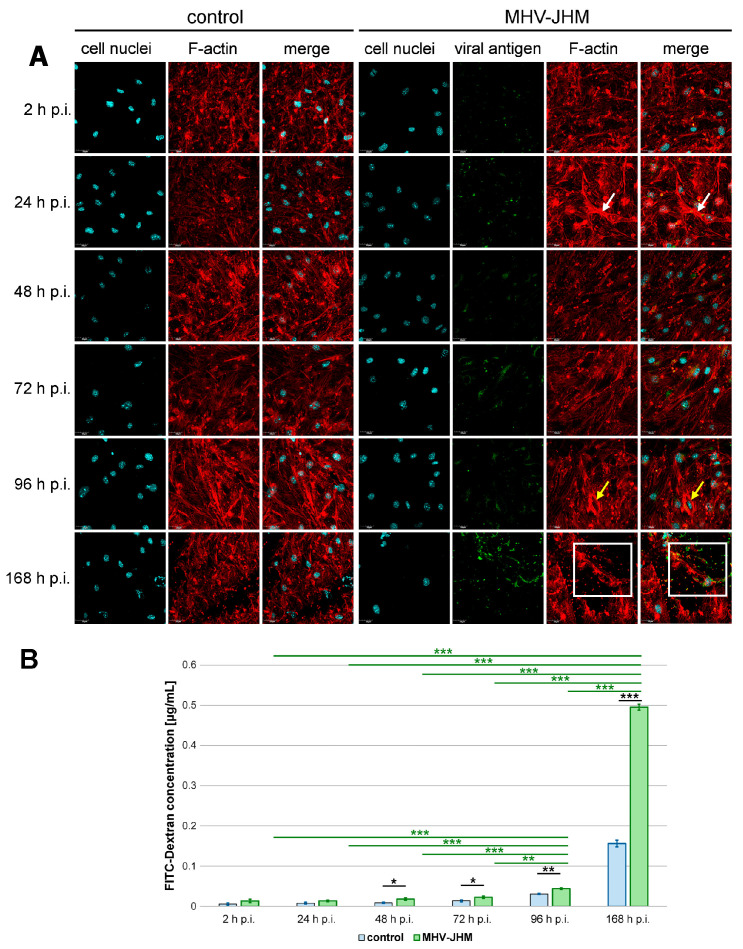
Representative confocal images of F-actin of bEnd.3 cells in the Transwell system (**A**) and the mean concentration of FITC-Dextran (µg/mL) that passed through the insert membrane (**B**). Each control co-culture was maintained for the same time (2–168 h p.i.—hour post-infection) as the corresponding infected co-culture. Blue—cell nuclei; red—F-actin; green—viral antigen; white arrows—F-actin condensation; yellow arrows—juxtanuclear ring; white square—viral antigen in the perinuclear space, polymerised F-actin (**A**). Scale 30 µm. Mean FITC-Dextran concentration is shown with SD (**B**). Kruskal–Wallis test with Dunn’s post hoc; * *p* < 0.05, ** *p* < 0.01, *** *p* < 0.001. Black asterisks—statistically significant changes in FITC-Dextran concentration in the receiver well between noninfected and infected co-culture at the given time post-infection. Green asterisks—statistically significant changes between infected co-cultures at different post-infection times.

A typical arrangement of F-actin into clamps was observed in the presented triple co-culture model, aligned with the insert membrane pores in uninfected and infected cells (Figure 3A). Both cortical actin rim structures and stress fibers were present in control and MHV-JHM infected cultures. No changes were detected at 2 h p.i. with MHV-JHM when compared to the control cells. However, at 24 h p.i., noticeable condensation of F-actin filaments was present (Figure 3A, white arrows). Interestingly, sudden decondensation was observed at 48 and 72 h p.i. A characteristic juxtanuclear ring formed at 96 h p.i.; within this structure, a higher presence of viral antigen signal was detected (Figure 3A, yellow arrows). Significant rearrangement and loss of the proper F-actin structure and local fragmentation occurred at 168 h p.i. The viral antigen signal was present in the perinuclear space, where the actin structure had been polymerised (Figure 3A, white squares).

At early time points, the amount of FITC-dextran that passed through infected monolayers was slightly elevated compared to controls but did not reach a statistical significance (0.006 ± 0.003 µg/mL (mean ± SD) in control vs. 0.013 ± 0.005 µg/mL in infected culture after 2 h, *p* = 0.128; 0.008 ± 0.002 µg/mL in control vs. 0.014 ± 0.002 µg/mL in infected culture after 24 h, *p* = 0.065). However, beginning at 48 h p.i., barrier permeability was significantly increased in infected cultures compared to controls (0.009 ± 0.002 µg/mL in control vs. 0.018 ± 0.003 µg/mL in infected culture, *p* = 0.036), and this trend continued at 72 h p.i. (0.014 ± 0.002 µg/mL in control vs. 0.022 ± 0.003 µg/mL in infected culture, *p* = 0.031) and 96 h p.i. (0.031 ± 0.002 µg/mL in control vs. 0.044 ± 0.002 µg/mL in infected culture, *p* = 0.003). The most pronounced effect was observed at 168 h p.i., where permeability was over three times higher than in the time-matched controls (0.156 ± 0.008 µg/mL in control vs. 0.495 ± 0.007 µg/mL in infected culture, *p* < 0.001) (Figure 3B).

To explore the temporal dynamics of barrier disruption in the infected cells, we compared FITC-dextran passage across time points within the infected group. Dextran passage at 168 h was significantly higher than at all earlier time points (0.013 µg/mL at 2 h p.i., *p* < 0.001; 0.0135 µg/mL at 24 h p.i., *p* < 0.001; 0.018 µg/mL at 48 h p.i., *p* < 0.001; 0.022 at 72 h p.i., *p* < 0.001; 0.044 at 96 h p.i., *p* < 0.001), with the mean concentration reaching 0.495 µg/mL. Significant increase in dextran passage was also observed at 96 h p.i. compared to 2 h p.i. (*p* < 0.001), 24 h p.i. (*p* < 0.001), 48 h p.i. (*p* < 0.001), and 72 h p.i. (*p* = 0.003) (Figure 3B).

### 3.3. High-Resolution Imaging of Morphological Changes in bEnd.3 Cell Line After Long-Term MHV-JHM Infection

**Figure 4:**
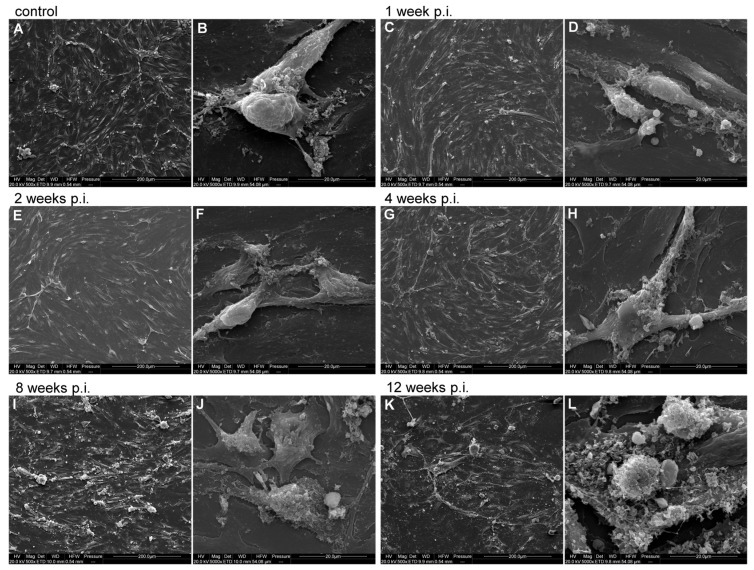
Representative SEM images of bEnd.3 cell line infected with MHV-JHM for 12 weeks. Control—uninfected cells; week p.i.—week post-infection. Cells at each time post-infection are shown in two magnifications: 500×, scale 200 µm (**A**,**C**,**E**,**G**,**I**,**K**) and 5000×, scale 20 µm (**B**,**D**,**F**,**H**,**J**,**L**).

Scanning electron microscopy (SEM) revealed progressive alterations in bEnd.3 cell morphology over 12 weeks of MHV-JHM infection when compared to uninfected control cells (Figure 4). A confluent monolayer of elongated, spindle-shaped endothelial cells with minimal gaps between them was observed in the control bEnd.3 culture (Figure 4A). Occasional small surface blebs were seen. At 5000× magnification (Figure 4B), the control cell surface was relatively smooth, with scattered protrusions and no significant debris. Adjacent cells remained closely apposed with no visible separation.

At 1 week p.i. with MHV-JHM (Figure 4C), the monolayer remained mostly intact and confluent, similar to the control. A few small intercellular gaps began to appear. Cells remained spread and elongated. At 5000× (Figure 4D), infected cells showed mild membrane ruffling and increased surface projections when compared to the control. Small protrusions were evident on the cell surface, indicating onset of cell stress. Interfaces between cells were still continuous with no obvious disruption. At 2 weeks p.i. (Figure 4E), subtle changes in cell distribution were observed—some cells appeared slightly retracted with less spread area, and inter-cell spacing increased slightly. Cellular debris (bright spots) was visible on the monolayer surface, suggesting some cell damage. Most cells remained attached, maintaining a near-confluent layer with only small gaps. At 5000× (Figure 4F), the cell surface exhibited more pronounced blebbing and roughness. Numerous surface blebs were present. Small gaps at cell borders were observed, though cells still contacted each other. At 4 weeks p.i. (Figure 4G), the monolayer showed noticeable irregularity. While still mostly confluent, there were regions of cell loss or thinning (darker patches between cells), indicating that some cells have detached. The remaining cells appeared elongated and irregularly distributed. At 5000× (Figure 4H), imaging revealed increased membrane activity—numerous protrusions covered the cell surface, and membrane ruffles were common along cell edges. Some aggregates of debris were visible on the surface, likely remnants of the lysed cells. Despite this, many cell–cell junctions remained intact, though there were early signs of separation (small clefts at cell borders). At 8 weeks p.i. (Figure 4I), a disrupted monolayer was observed. Cells no longer formed a continuous monolayer. There were larger gaps where cells had detached. The remaining cells clustered, leaving open spaces between the groups of cells. Individual cells appeared smaller and rounder than in earlier weeks, with some clustering around debris. At 5000× (Figure 4J), cell surfaces were highly irregular and rough. Extensive membrane blebbing was observed. Cells were covered in debris. Notably, junctional gaps between neighbouring cells were visible. Cell membranes at the edges were ruffled and folded, indicating significant structural damage. At 12 weeks p.i. (Figure 4K), the most severe alterations were observed. The cell monolayer was largely deteriorated—many areas were devoid of intact cells, covered only by cellular debris (white, fragmented material). Surviving cells were often isolated, retracted and small, leaving extensive intercellular gaps. At 5000× (Figure 4L), the cell surface was covered with granular debris and collapsed cell fragments. Cells had highly blebbed surfaces and were presented as rough spheres. No continuous contact between cells was seen, consistent with the breakdown of the endothelial monolayer.

### 3.4. Progressive Mitochondrial Network Remodeling During Long-Term MHV-JHM Infection

**Figure 5:**
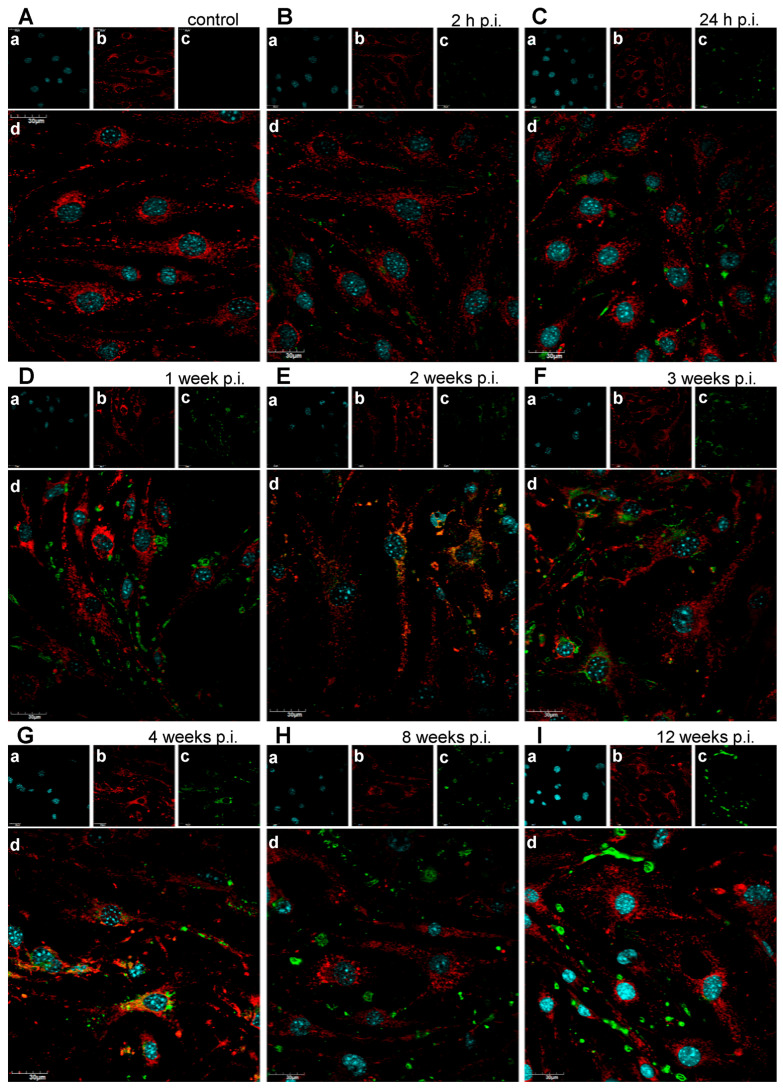
Representative confocal images of mitochondria of uninfected bEnd.3 cells (**A**) and bEnd.3 cells infected with MHV-JHM (**B**–**I**). h p.i.—hour post-infection; week p.i.—week post-infection. Blue—cell nuclei (**a**); red—mitochondria (**b**); green—viral antigen (**c**); merged image (**d**). Magnification 60×. Scale 30 µm.

Confocal imaging of control bEnd.3 cells revealed well-organised, elongated, and interconnected mitochondrial networks distributed throughout the cytoplasm, with no detectable viral antigen. At 2 h p.i. with MHV-JHM, mitochondrial morphology remained largely comparable to control, though a subtle increase in mitochondria number was visible. Viral antigen (S protein) was detectable in a subset of cells, appearing as small, punctate green foci within the cytoplasm, often located near mitochondrial clusters. By 24 h p.i., mitochondrial fragmentation became more pronounced, with a higher number of individual mitochondria and a corresponding reduction in large interconnected networks. Viral antigen staining intensified and appeared in close spatial proximity to the fragmented mitochondria, suggesting potential mitochondrial association or redistribution in infected cells. Between 1 and 4 weeks p.i., viral antigen was abundant, frequently appearing as perinuclear or cytoplasmic clusters overlapping or adjacent to areas of mitochondrial fragmentation. By 8 weeks p.i., mitochondrial networks were severely disrupted, with predominantly punctate mitochondria and dense, aggregated clusters, while viral antigen persisted in perinuclear regions. At 12 weeks p.i., dense accumulations of mitochondria were observed, often co-occurring with viral antigen staining (Figure 5).

To evaluate how long-term MHV-JHM infection alters mitochondrial morphology in murine brain endothelial cells, we quantified mitochondrial network features using ImageJ with the MiNA analysis tool. Confocal images of 100 bEnd.3 cells per each time point were subjected to the analysis. Parameters analysed included the number of individual mitochondria, number of mitochondrial networks, individual-to-network ratio, mean branch length [µm], mean network size (branch number), and mitochondrial footprint [µm^2^]. 

**Figure 6:**
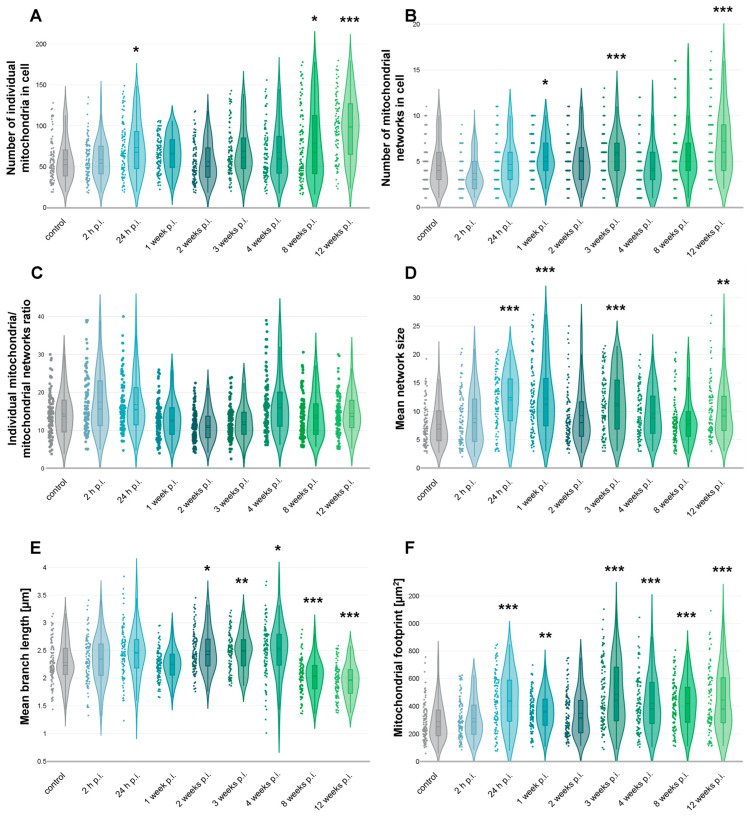
Mitochondrial network analysis based on confocal images of bEnd.3 cell line infected with MHV-JHM. Graphs showing number of individual mitochondria in cell (**A**), number of mitochondrial networks in cell (**B**), ratio of individual mitochondria to mitochondrial networks in cell (**C**), mean size of mitochondrial network (**D**), mean branch length [µm] (**E**), and mitochondrial footprint [µm^2^] (**F**). Dots, violin plots and box plots showing the distribution of data across time post-infection. The dashed line in the box plot inside a violin plot—the mean value; the straight line is the median value. Kruskal–Wallis test with Dunn’s post hoc; * *p* < 0.05, ** *p* < 0.01, *** *p* < 0.001.

Quantitative analysis confirmed earlier qualitative observations. The number of individual mitochondria per cell increased across the infection timeline. While there were no significant differences between control and 2 h p.i. (*p* = 1.0), an increase was statistically significant at 24 h p.i. (*p* = 0.034 vs. control). No significant changes were detected at 1–4 weeks p.i.; however, the change in number of individual mitochondria became significant at 8 weeks (*p* = 0.047) and 12 weeks p.i. (*p* < 0.001). The number of individual mitochondria reached 98.4 ± 37.7 (mean ± SD) at 12 weeks p.i., nearly doubling the control value of 58.5 ± 25.9 (Figure 6A).

Interestingly, the number of mitochondrial networks also increased over time, particularly at later time points following infection. Although there were no significant differences between control and 24 h or 2 weeks p.i., a significant increase in network number was observed at 1 week p.i. (*p* = 0.036 vs. control), 3 weeks p.i. (*p* = 0.008), and at 12 weeks p.i. (*p* < 0.001), where the average number of networks per cell increased, respectively, to 5.49 ± 2.11, 5.75 ± 2.51, and 7.2 ± 3.6 compared to 4.5 ± 2.4 in controls (Figure 6B).

The individual-to-network ratio decreased from 15.0 ± 7.6 in controls to 12.5 ± 6.3 at 2 weeks p.i. but was not statistically significant. By 4 weeks p.i., although not significantly, the ratio increased to 20.3 ± 16.7, suggesting re-fragmentation or re-distribution of mitochondria into more isolated states (Figure 6C).

Mean network size (branches per network) analysis revealed significant changes at 24 h p.i. (*p* < 0.001 vs. control), 1 week p.i. (*p* < 0.001), 3 weeks p.i. (*p* < 0.001), and 12 weeks p.i. (*p* = 0.007). The size rose from 7.68 ± 3.6 branches per network in control cells to 12 ± 4.85, 12.22 ± 5.97, 11.42 ± 5.18, and 10.06 ± 4.4 branches per network, respectively (Figure 6D).

Mean branch length remained relatively stable during early times post-infection but showed changes at the later stages. Significant changes were observed at 2 weeks p.i. (*p* = 0.023 vs. control), 3 weeks p.i. (*p* = 0.01) and 4 weeks p.i. (*p* = 0.003), where mean branch length increased from 2.28 ± 0.38 µm in control cells to 2.48 ± 0.37 µm, 2.48 ± 0.34 µm, and 2.5 ± 0.44 µm, respectively. Later, at 8 weeks p.i. (*p* < 0.001) and 12 weeks p.i. (*p* < 0.001), a drop in mean branch length was observed (2.03 ± 0.32 and 1.95 ± 0.30 µm, respectively) (Figure 6E).

Total mitochondrial area per cell significantly changed at 24 h p.i. (*p* < 0.001 vs. control), 1 week p.i. (*p* = 0.007), 3 weeks p.i. (*p* < 0.001), 4 weeks p.i. (*p* < 0.001), 8 weeks p.i. (*p* < 0.001), and 12 weeks p.i. (*p* < 0.001). The area increased from 283.4 ± 125.1 µm^2^ in control cells to 433.2 ± 188.4, 364.4 ± 128.6, 482.2 ± 221.7, 418 ± 190.9, 419.5 ± 166.4, and 443 ± 222.4 µm^2^, respectively (Figure 6F).

### 3.5. Long-Term MHV-JHM Infection Induces Rapid and Sustained ROS Production in bEnd.3 Cells

**Figure 7:**
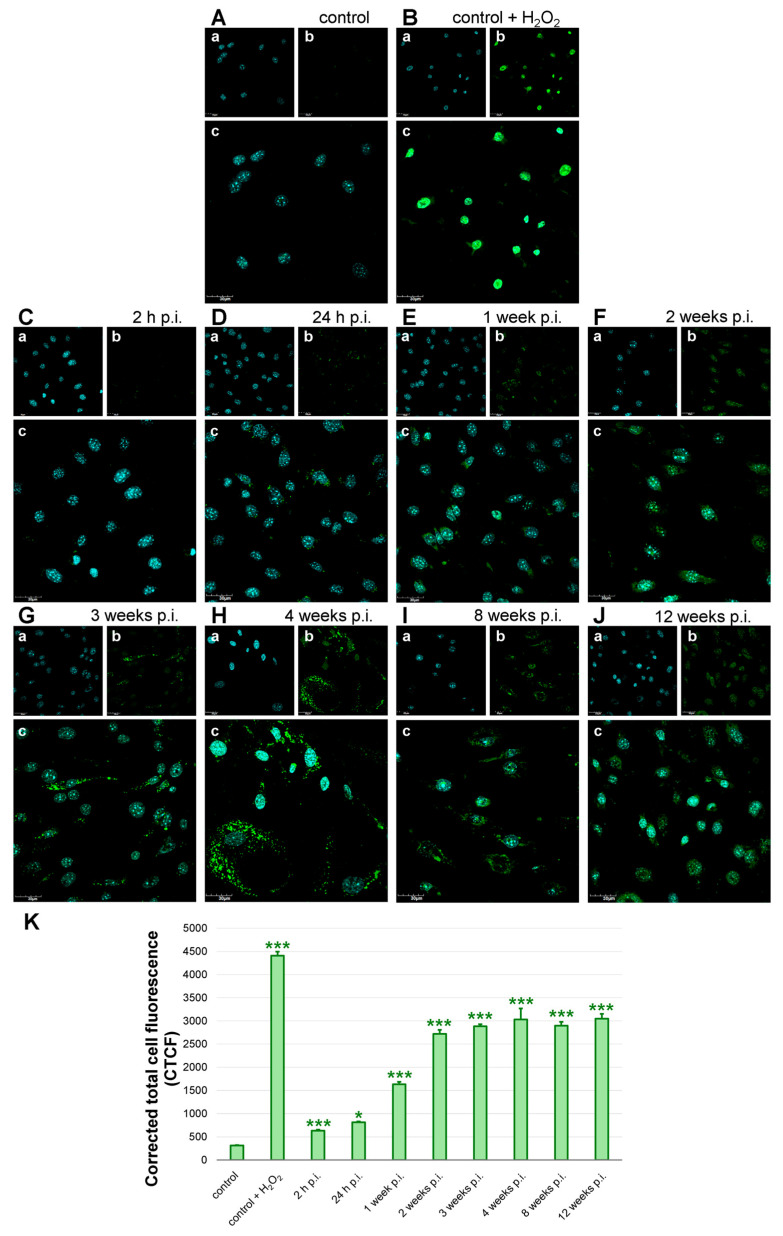
ROS production in bEnd.3 cell line after long-term MHV-JHM infection. Control—uninfected cells (**A**); control + H_2_O_2_—positive control, uninfected cells treated with H_2_O_2_ before staining (**B**); infected cells (**C**–**J**). Analysis of CTCF corresponding to ROS levels in bEnd.3 cells (**K**). h p.i.—hour post-infection; week p.i.—week post-infection. Blue—cell nuclei (**a**); green—ROS (**b**); merged image (**c**). Magnification 60×. Scale 30 µm (**A**–**I**). ROS levels were shown as mean CTCF with SE. Kruskal–Wallis test with Dunn’s post hoc; * *p* < 0.05, *** *p* < 0.001 (**K**).

Morphological analysis of ROS fluorescence revealed that ROS levels were scarce in control, uninfected cells (Figure 7A). In control, uninfected cells treated with H_2_O_2_ high levels of green fluorescence signal were detected (Figure 7B). In early time points post-infection, ROS fluorescence appeared as a diffuse cytoplasmic staining, while in later stages it formed intense perinuclear and punctate clusters (Figure 7C–J). As assessed by corrected total cell fluorescence (CTCF) from green fluorescence imaging, quantitative analysis showed a marked and statistically significant elevation following MHV-JHM infection compared to uninfected controls (Figure 7K). In the uninfected cells, baseline ROS production was low at 312.9 ± 10.6 (mean ± SE), while treatment with H_2_O_2_, used as a positive control, produced a robust increase to 4407.3 ± 86.6 (*p* < 0.0001 vs. control), validating the assay’s sensitivity. At 2 h p.i., a modest but significant rise in ROS was observed (631.5 ± 22.4, *p* < 0.0001), followed by a further increase at 24 h p.i. (813.0 ± 19.0, *p* = 0.048). These early time points suggest that ROS production was rapidly induced following viral exposure. A pronounced increase was seen at 1 week p.i., with mean CTCF reaching 1635.0 ± 51.6 (*p* < 0.0001). ROS levels continued to increase at 2 weeks p.i. to 2722.5 ± 83.1 (*p* < 0.0001) and remained significantly elevated through 3, 4, 8, and 12 weeks p.i. at 2886.6 ± 40.2 (*p* < 0.0001), 3031.3 ± 237.6 (*p* < 0.0001), 2896.2 ± 82.0 (*p* < 0.0001), and 3049.4 ± 102.4 (*p* < 0.0001), respectively. Values obtained at 4–12 weeks p.i. were comparable to the H_2_O_2_-treated group, with no significant differences detected (*p* > 0.05), indicating a persistent, high level of oxidative stress in long-term infected cultures.

## 4. Discussion

The blood–brain barrier (BBB) is essential for maintaining central nervous system (CNS) homeostasis, and its disruption is increasingly recognised as a contributing factor in both acute and chronic neurological diseases [54,55]. Viral infections of the CNS can compromise BBB integrity through multiple mechanisms, ranging from direct effects on endothelial cells to secondary oxidative and inflammatory cascades [56,57,58,59,60,61]. While many studies have focused on the acute impact of neurotropic viruses on BBB function, less is known about the consequences of prolonged or persistent infection, particularly in the context of chronic viral presence. In this study, we examined the effects of long-term infection with the neurotropic murine coronavirus strain MHV-JHM on brain endothelial cells using the bEnd.3 cell line. Our results revealed a multifaceted and progressive deterioration of endothelial barrier integrity, driven by mitochondrial dysfunction, oxidative stress, and ultrastructural cell damage.

Although immortalised brain endothelial models exhibit lower absolute tightness than primary or induced pluripotent stem cells (iPSC)-derived BBB systems, bEnd.3 retains essential junctional, transport, and signalling features, enabling sensitive within-model detection of paracellular flux changes and cytoskeletal/ultrastructural injury. Studies established bEnd.3 tight-junction protein expression and functional barrier formation suitable for permeability assays [62,63]. bEnd.3 cell line also responds robustly to pathophysiological stimuli central to BBB failure—pro-inflammatory cytokines, angiotensin II, and the oxidative stress [64,65,66]. While iPSC-BMECs offer a markedly higher transepithelial/transendothelial electrical resistance (TEER), they require lengthy differentiation and can show mixed endothelial–epithelial signatures, while bEnd.3 cell line stability and scalability makes it well-suited for probing progressive, long-term effects, as shown in our 12-week MHV-JHM infection model [67,68,69].

Our BBB co-culture model combined endothelial cells (bEnd.3), astrocytes (C8D1A), and neuroblasts (Neuro-2a), and was maintained for up to one week. Chosen cell lines can be cultured in the same medium, ensuring reproducibility and stability across permeability assays. While this simplified tri-culture reproduces key features of the NVU, we acknowledge that it does not include microglia or pericytes, critical modulators of BBB physiology. Pericytes regulate tight junction expression, vascular stability, and vesicular trafficking, whereas microglia interact with the vasculature and astrocytic end-feet, exerting both protective and disruptive effects depending on the inflammatory context [70,71]. Because of culture compatibility constraints, we did not include additional NVU cell types, which would require specialised conditions that are not easily adapted to insert-based short-term assays. Simplified co-cultures of endothelial cells with astrocytes and neurons have been used previously to study barrier function, viral infection, and drug transport [50,68,72,73]. Future work using more complex organoid or microfluidic NVU platforms could expand upon our findings.

One of the changes observed in MHV-JHM–infected bEnd.3 cells was the progressive fragmentation of the mitochondrial network (Figure 6). Quantitative image analysis revealed a near-doubling in the number of individual mitochondria per cell by 8–12 weeks post-infection (Figure 6A), with corresponding decreases in average branch length (Figure 6E). This shift from elongated, interconnected networks toward numerous punctate mitochondria is a hallmark of mitochondrial fission and dysfunction. Excessive mitochondrial fission is often driven by activation of Drp1 and related fission machinery, especially under sustained stress. Fragmented mitochondria tend to produce ROS and release pro-apoptotic factors, creating a feed-forward cycle of cellular stress [74,75,76,77,78]. Endothelial cells experiencing inflammatory or oxidative stress have been shown to undergo Drp1-mediated mitochondrial fragmentation, which in turn can impair BBB properties [12,79]. In our model, long-term coronavirus infection likely triggers mitochondrial fission through direct viral manipulation of host organelles or secondary factors like calcium and ROS signalling. 

The infected endothelial cells showed a significant increase in the total mitochondrial area per cell despite fragmentation (Figure 6F), suggesting possible compensatory mitochondrial biogenesis or swelling of damaged mitochondria. However, accumulation of swollen, dysfunctional mitochondria can be detrimental, as impaired mitophagy fails to clear these ROS-generating organelles [80,81,82]. Consistent with this, the long-term infected cultures endured a high oxidative burden (Figure 7). From a broader perspective, the mitochondrial changes observed here provide a potential mechanistic link between viral infection and endothelial dysfunction. Many viruses hijack or damage mitochondria to evade immune responses; coronaviruses in particular are known to alter mitochondrial dynamics and bioenergetics as part of their pathogenesis [83,84,85,86]. The mitochondrial dysfunction we report could be one upstream driver of BBB failure, highlighting a novel aspect of coronavirus neuropathogenesis.

It is important to note that mitochondrial remodelling under long-term MHV-JHM infection did not follow a strictly linear trajectory. While some parameters, such as the number of individual mitochondria (Figure 6A), showed a gradual and significant increase over time, other features, including mean network size (Figure 6D) and mean branch length (Figure 6E), displayed fluctuations at intermediate time points. Such variability is consistent with previous studies reporting that mitochondrial dynamics are highly responsive to the changing balance between fission, fusion, and mitophagy during cellular stress and viral infection [87]. For coronaviruses specifically, several viral proteins directly target mitochondria, leading to fragmentation, disrupted dynamics, and excessive ROS generation [88,89,90,91]. These alterations are not necessarily linear: transient increases in network connectivity may reflect compensatory fusion events aimed at preserving mitochondrial function, which are later overcome by dominant fission and fragmentation under persistent stress. Therefore, the apparent inconsistencies in Figure 6 should be interpreted not as methodological unreliability, but rather as the evidence of dynamic, adaptive nature of mitochondrial networks in response to long-term coronavirus infection.

Long-term MHV-JHM infection induced a robust and sustained increase in intracellular ROS levels in bEnd.3 cells. We detected a rapid rise in ROS as early as 24 h post-infection, and by 1–2 weeks post-infection, ROS levels had surged to nearly ten-fold above baseline, remaining almost as high as H_2_O_2_-treated positive controls through 12 weeks (Figure 7). This prolonged, extensive oxidative stress is a clear indicator of redox imbalance in the infected endothelial cells. There are several plausible sources for the heightened ROS. One is NADPH oxidase activation: pro-inflammatory cytokines or viral components can stimulate Nox enzymes in endothelial cells, leading to superoxide and peroxide generation. In the context of viral infection, pattern recognition receptors (e.g., TLRs) and downstream signals (e.g., NF-κB) could upregulate Nox isoforms or associated subunits, as has been shown for other viruses and inflammatory stimuli [92,93]. Another major source of ROS is the dysfunctional mitochondria discussed earlier. Fragmented mitochondria often leak electrons and produce excessive ROS (mitoROS) due to impaired electron transport chain function [94]. Coronaviruses are known to trigger an increase in cytosolic and mitochondrial ROS in host cells by enhancing ROS production and dampening antioxidant defences [95,96]. Our long-term infected cultures likely suffer from both mechanisms: an initial ROS burst from acute infection and a sustained phase driven by accumulating mitochondrial damage. The consequences of chronic ROS elevation in the brain endothelium are deleterious for the BBB. ROS can directly oxidise and damage tight junction proteins and adherens junction components, leading to their dissociation or degradation [97,98]. Elevated ROS also activate matrix metalloproteinases that can cleave extracellular matrix and junctional proteins, as noted in MHV-JHM infections in vivo [99]. In our study, the later time points showing persistent high ROS coincided with structural degeneration of the endothelial monolayer (membrane blebbing, cell detachment) (Figure 4). This temporal correlation supports the idea that oxidative stress is a driving force behind barrier disruption. In viral infections, oxidative stress has been proposed to mediate much endothelial damage [96,100]. Notably, the ROS increase in MHV-JHM–infected cells also has implications for antiviral responses and viral persistence. Moderate ROS production can activate antiviral signalling pathways, but excessive ROS may impair antiviral immunity [101]. The high ROS levels observed in our long-term infection model could indicate a state of endothelial oxidative injury that harms the BBB endothelial cells and potentially allows viral persistence by subverting adequate immune clearance.

Scanning electron microscopy depicted the structural deterioration that unfolded in the infected endothelial monolayer over time (Figure 4). While control bEnd.3 cells formed a confluent layer of tightly apposed cells with smooth surfaces, and infected cultures gradually lost this orderly architecture. By 4–8 weeks post-infection, SEM images showed infected endothelial cells becoming irregularly shaped and withdrawing from one another, with increasing intercellular gaps. The cell surfaces in infected samples turned from relatively smooth to rough and blebbled, covered in numerous protrusions and patches of particulate debris. By 12 weeks, the ultrastructural damage was profound—large areas of the culture had lost intact cells, leaving behind membranous debris, and the remaining cells were shrunken and presented as large spheres with highly blebbed surfaces. Notably, cell–cell junctions were no longer discernible at that stage. This progression of morphological damage—from subtle surface ruffling at 1–2 weeks to complete monolayer collapse by 3 months—highlights the cumulative cytopathic effect of long-term MHV-JHM infection on endothelial cells. The SEM findings corroborate and extend our fluorescence-based observations (mitochondria, ROS) by showing the result of the intracellular stress on the physical integrity of the endothelial layer. It is likely that tight junction complexes (e.g., ZO-1, claudin-5) and adherens junctions (VE-cadherin) were degraded or internalised during this period, leading to the loss of adhesion seen ultrastructurally. This is consistent with the reports from acute MHV-3 infection, where ZO-1 and VE-cadherin were lost from endothelial borders shortly after infection [46]. Our SEM images confirm that long-term viral infection can erode the endothelial monolayer structure. Several factors likely underlie observed ultrastructural damage. Proteases, such as MMPs secreted by infected cells, can digest extracellular matrix and undermine the adhesion of cells to the basement membrane, facilitating detachment [102,103,104]. For instance, in West Nile virus infection, MMP-9 produced by astrocytes was shown to degrade endothelial basal lamina, contributing to BBB disruption [105,106]. It is plausible that MHV-JHM infection induced MMPs in our culture, gradually breaking down adhesive substrates or junctional proteins. A recent study using primary human in vitro BBB models has shown that components of the SARS-CoV-2 spike protein, including S1, S2 and the receptor binding domain, can all induce BBB leakage without toxicity. Induction of BBB leakage occurred in the response to glycosylated and non-glycosylated forms of S1 and S2 [107]. Infection of primary human endothelial cells overexpressing ACE2 with SARS-CoV-2 induced the overexpression of coagulation factors, adhesion molecules and pro-inflammatory cytokines, as well as the formation of multinucleated syncytia and endothelial cell lysis [108]. It is worth noting that the increased permeability of the blood–brain barrier leads to blood infiltration and the influx of plasma proteins, including fibrinogen. As its accumulation in the brain tissue is associated with many neurodegenerative diseases, it is a marker for studying barrier structure in disease states. Changes also occur in other cellular elements of the nervous system, including the axons and Schwann sheath, as well as microglia and oligodendrocytes [109]. In addition, neuronal swelling caused by chronic inflammation and increased intracranial pressure also adds to the nervous system dysfunction [4]. In summary, the ultrastructural alterations we documented provide a visual confirmation that the BBB model is functionally compromised. The loss of cell–cell contacts and extensive cell debris by the end of the experiment underscores that long-term viral presence eventually overcomes the endothelial cells’ defences. This lends support to the notion that chronic viral infections, even if non-cytolytic initially, can lead to the eventual vascular unit degeneration.

In the co-culture model, we observed pronounced remodelling of the F-actin cytoskeleton in infected endothelial monolayers (Figure 3A). Within days of MHV-JHM infection, phalloidin staining revealed that bEnd.3 cells transitioned from the typical actin arrangement in control cells to enhanced actin condensation at 24 h p.i. At 48 and 72 h p.i. decondensation of actin filaments was observed. Alterations became more extensive by 96 and 168 h p.i., where cells exhibited juxtanuclear rings, and intercellular gaps formed in the monolayer. Such cytoskeletal changes are a well-known response of endothelial cells to stress and are tightly linked to barrier dysfunction. Coronavirus infection is associated with cytoskeletal changes. Our team previously demonstrated that MHV-JHM can manipulate the cytoskeleton of primary murine neurons during a one-week infection. Furthermore, MHV-JHM uses tunnelling nanotubes to transport itself from cell to cell, thus avoiding the immune response and direct receptor binding [110]. Studies on SARS-CoV have reported actin reorganisation in infected cells, potentially through viral NSPs and accessory proteins that hijack actin motors for transport or induce filopodia formation [111,112,113,114,115]. The remodelling of F-actin we observed is significant for BBB integrity. By 168 h p.i., the actin cytoskeletal disruption coincided with a 3-fold increase in FITC-dextran permeability across the bEnd.3 monolayer, underscoring the functional consequence of observed cytoskeletal breakdown.

Using a FITC-Dextran assay in a triple co-culture BBB model, we demonstrated that MHV-JHM infection induces time-dependent increases in paracellular leakage (Figure 3B). In the early infection stages (2–24 h), we noted trends toward higher dextran flux in the infected monolayers, though not immediately significant. However, by 2–3 days post-infection, permeability in the infected cultures became significantly elevated compared to uninfected controls, and this difference increased with time. At 7 days post-infection (168 h), the dextran concentration in the receiver chamber was over three times higher in infected co-cultures than in the time-matched controls, indicating a profound loss of barrier tightness. Importantly, we also found that permeability in the infected group at 7 days was significantly higher than at any earlier time point during the infection, confirming a progressive deterioration of barrier function over the course of a week. These results complement our long-term observations (up to 12 weeks) qualitatively, as by those later times the barrier was collapsing (though we did not quantify dextran beyond 7 days, the monolayer integrity, visualised using SEM, was severely compromised by weeks 8–12). The permeability assay underscores that even in a model incorporating supporting astrocytes, the endothelium cannot maintain its selective barrier function when infected with MHV-JHM. The increase in FITC-dextran flux suggests enlargement of inter-endothelial spaces and/or loss of tight junction selectivity, allowing a 70 kDa molecule to cross relatively freely between compartments. Our in vitro findings are in line with in vivo evidence of coronavirus-induced BBB disruption. As mentioned before, a virulent MHV strain caused enhanced BBB permeability in mice, correlating with reduced levels of tight junction proteins [46]. Clinically, patients with severe COVID-19 have been reported to have elevated BBB permeability markers and imaging signs of barrier leakage, which is analogous to our observation that coronavirus infection can compromise barrier function [25,116].

Taken together, our findings paint a comprehensive picture of how a long-term coronavirus infection can methodically erode the blood–brain barrier’s integrity. These changes likely operate in concert to compromise the BBB’s two primary roles: forming a physical tight seal and serving as a metabolic regulatory interface. This has significant implications for viral neuroinvasion. A weakened BBB can facilitate the entry of the virus directly into the CNS. It also allows peripheral immune cells and cytokines to flood the CNS, which can either help clear the virus or cause a neuropathology. Our study suggests that even without systemic inflammation, a virus confined to the endothelial layer can, over time, generate a microenvironment of oxidative stress and cellular dysfunction that leads to barrier failure. This mechanism might be particularly relevant in persistent viral infection or reactivation scenarios—for example, subclinical chronic infections that slowly degrade vascular health and could contribute to long-term neurological syndromes. There has been speculation that post-viral syndromes might involve residual virus in tissues, causing ongoing oxidative and inflammatory damage [117,118,119,120,121]; our results with a model betacoronavirus lend credence to the idea that persistent virus in the vasculature could lead to enduring BBB impairment and thus chronic neurological symptoms.

While we recognise the limitations of the bEnd.3-based system, these results provide mechanistic evidence that coronavirus infection can compromise BBB-like properties through mitochondrial dysfunction and oxidative stress. Future work using more complex models incorporating pericytes and microglia and in vivo validation will be necessary to confirm the full impact of coronavirus infection on BBB physiology.

## 5. Conclusions

In this study, we examined the effects of persistent MHV-JHM coronavirus infection on the in vitro BBB model. Our findings demonstrate that infection of brain endothelial cells leads to the (i) progressive remodeling of mitochondrial networks, consistent with increased fission and reduced connectivity; (ii) sustained ROS overproduction, suggesting oxidative stress as a central driver of endothelial dysfunction; (iii) increased paracellular permeability, as assessed by FITC-dextran flux across confluent monolayers; and (iv) ultrastructural abnormalities, including a loss of smooth surface morphology and disruption of cell–cell junctions.

Our study provides an analysis of how a long-term MHV-JHM infection impacts brain endothelial cells, revealing a cascade of structural and functional derangements that culminate in BBB disruption. These findings contribute to a deeper understanding of coronavirus-associated neuropathogenesis, highlighting that the BBB endothelium is both a target and mediator of viral injury. Given the parallels between MHV and human neurotropic coronaviruses, these insights may advise future investigations into viral encephalopathies and inspire strategies to protect the BBB, thereby safeguarding the brain from both virus and immune-mediated damage during prolonged infections. The BBB is a critical line of defence in the CNS; our work underscores that when a virus undermines that line from within, the effects can be far-reaching for neural homeostasis. Protecting or restoring BBB function in the face of viral infection could thus be key to preventing long-term neurological sequelae.

## Data Availability

All original contributions presented in this study are included in this article. Further inquiries can be directed to the corresponding authors.
